# *Trametes* sp. as a Source of Biopolymer Cross-Linking Agents: Laccase Induced Gelation of Ferulated Arabinoxylans

**DOI:** 10.3390/molecules14104159

**Published:** 2009-10-16

**Authors:** Alva Castillo, Agustín Rascón-Chu, Georgina Vargas, Elizabeth Carvajal-Millán, Elisa Valenzuela-Soto, Rogerio R. Sotelo-Mundo, Ana Luisa Martínez

**Affiliations:** 1 Facultad de Zootecnia, Universidad Autónoma de Chihuahua, Chihuahua, Chihuahua 31120, Mexico; 2 Research Center for Food and Development, CIAD, A. C, DTAOV, Hermosillo, Sonora 83000, Mexico; 3 Research Center for Food and Development, CIAD, A. C, DTAOA, Hermosillo, Sonora 83000, Mexico; 4 Research Center for Food and Development, CIAD, A. C, DCA, Hermosillo, Sonora 83000, Mexico

**Keywords:** polysaccharide, cross-linking, ferulic acid, viscosity, gels

## Abstract

This study examined the feasibility of using *Trametes* sp. grown under drought conditions to catalyze the covalent cross-linking of ferulated arabinoxylans. The rate of polymerization of ferulated arabinoxylans solutions at 0.5% (w/v) was dose dependent on the laccase concentration in the system. Above 1.2 nkat laccase/mg ferulated arabinoxylan, the rate of cross-linking reached a plateau, suggesting that the reaction reached saturation. For 2% (w/v) ferulated arabinoxylans treated with laccase (1.6 nkat/mg ferulated arabinoxylan), stable gels were formed after 4 h at 25 °C, with cross-linking contents (diferulic and triferulic acid) contents of 0.03 and 0.015 μg/mg ferulated arabinoxylan, respectively. This study demonstrated that *Trametes* sp. can be a source of biopolymer cross-linking enzymes like laccase.

## 1. Introduction

Basidiomycetes white-rot fungi efficiently degrade lignin [1]. They have a powerful extracellular enzymatic complex, able to depolymerize this aromatic polymer into lower molecular weight compounds [2]. White-rot fungi metabolic activity and cell growth depend on environmental conditions and specific enzymatic regulation mechanisms, which are very important for enzyme production. These organisms need to excrete digestive enzymes for absorption of organic materials as a way of surviving environmental variations which induce specific changes in growth and development patterns as a fungus response [3]. Laccases (benzenediol: oxygen oxidoreductases; EC 1.10.3.2.) are glycosylated polyphenol oxidases containing four copper ions per molecule, which are produced by white rot fungi [4]. The genus *Trametes* includes many white-rot fungi species, and several produce these enzymes in large amounts [5]. Laccases have widespread applications such as effluent decolouration, pulp bleaching, removal of phenolics from wines, organic synthesis, biosensors, synthesis of complex medical compounds, among others. Laccases are also able to cross-link biopolymers containing phenolic acids like ferulated arabinoxylans [6]. Ferulated arabinoxylans are polysaccharides located mainly in the cell wall of cereals, formed by links β(1→4)-D-xylose branched with α(1→3)-L-arabinose. Some of the arabinose residues contain ester-linked ferulic acid [7]. Laccase can oxidize ferulic acid present in arabinoxylans resulting in the formation of a covalent gel stabilized by dimers and a trimer of ferulic acid [6,8,9]. Enzymatic cross-linking of food biopolymers is receiving increasing attention as this enables modification of structural properties of the food matrix [10]. In addition, enzymatic cross-linking and grafting of specific substances to the biopolymers can be exploited in food and nonfood applications allowing for generation of novel biomaterials [11]. The objective of this study was to evaluate the feasibility of covalent cross-link ferulated arabinoxylans applying a crude extract of *Trametes* sp. that grows commonly in Northern Mexico under drought conditions as a novel non expensive source of biopolymers cross-linking agents like laccase. 

## 2. Results and Discussion

### 2.1. Laccase activity

Supernatant from *Trametes* sp. was assayed for laccase activy. Laccase specific activity in units per mg protein reached a maximum at the 7^th^ day and then a reduction was observed. Nonetheless, the laccase biomass continued to increase. These results are similar to that reported for *Trametes versicolor*, which presents its maximum laccase activity on the third day and the mycelium continues to develop [12]. This experiment is critical to obtain maximum enzyme yields for future scale-up processes.

### 2.2. Laccase induced cross-linking of ferulated arabinoxylans

A dose-depentent ferulated arabinoxylans polymerization was observed using 0.2 to 1.7 nkat/mg laccase, reflected in an increment in the specific viscosity of 0.5% (w/v) ferulated arabinoxylans solutions (Figure 2a). The increase in viscosity reflected the formation of covalent linkages between ferulic acid residues of adjacent arabinoxylan chains. In Figure 2b the slope (cross-linking rate) of the initial linear increase of specific viscosity was represented as a function of enzyme concentration (nkat laccase/mg ferulated arabinoxylans). The rate of cross-linking was clearly related to the laccase content in the system. Above 1.2 nkat laccase/mg ferulated arabinoxylans, no significant increase in the rate of cross-linking was registered, suggesting that after this laccase amount the ferulated arabinoxylans cross-linking reaction is developed under enzyme excess conditions.

**Figure 1:**
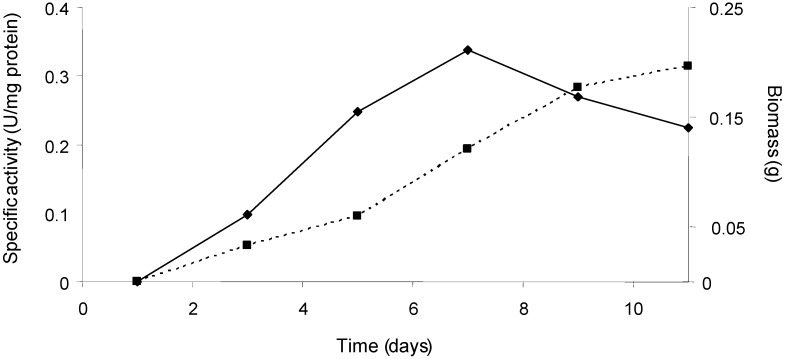
Relationship between biomass (dotted line) and specific activity (continuous line) of the enzyme laccase produced by *Trametes* sp.

In 2% (w/v) solutions of ferulated arabinoxylans treated with laccase (1.6 nkat/mg ferulated arabinoxylans) stable gels were formed after 4 h of incubation at 25 °C (the system did not deform under its own weight when the vessel containing the reaction mixture was tilted). At the end of gelation, 75% of the ferulic acid (FA) initially present in the ferulated arabinoxylans was oxidized, while only 17% of oxidized FA was recovered as di- and tri-FA (Table 1). In fact, the di- and tri-FA content in ferulated arabinoxylans did not increase after laccase-induced gelation, but rather they decreased from 0.77 to 0.02 and from 0.39 to 0.01 μg/mg ferulated arabinoxylans. This decrease in FA content without a proportional formation of di- and tri-FA structures has been also reported in wheat arabinoxylan gels induced by a laccase from *Pyconoporus cinnabarinus* [6] and in maize heteroxylan gels formed by a peroxidase/H_2_O_2_ system [13]. The explanation for this phenomenon has been related to a hypothesis of formation of non reported di- and tri-FA or higher ferulate structures and/or to physical interactions between arabinoxylan chains. Furthermore, in the present study, the relative percentages of di-fa structures (isomeric forms of di-FA) found in ferulated arabinoxylans gels were different to those found in the ferulated arabinoxylans before gelation. In gelled ferulated arabinoxylans only the 5-5´ and 8-*O*-4´ di-FA isomeric structures were detected at relative percentages of 50% while in ferulated arabinoxylans before gelation the relative percentages of each di-fa structure were: 16, 21 and 63% for the 8-5´(mainly in the benzofuran form), 8-*O*-4´ and 5-5´ structures, respectively.

**Figure 2:**
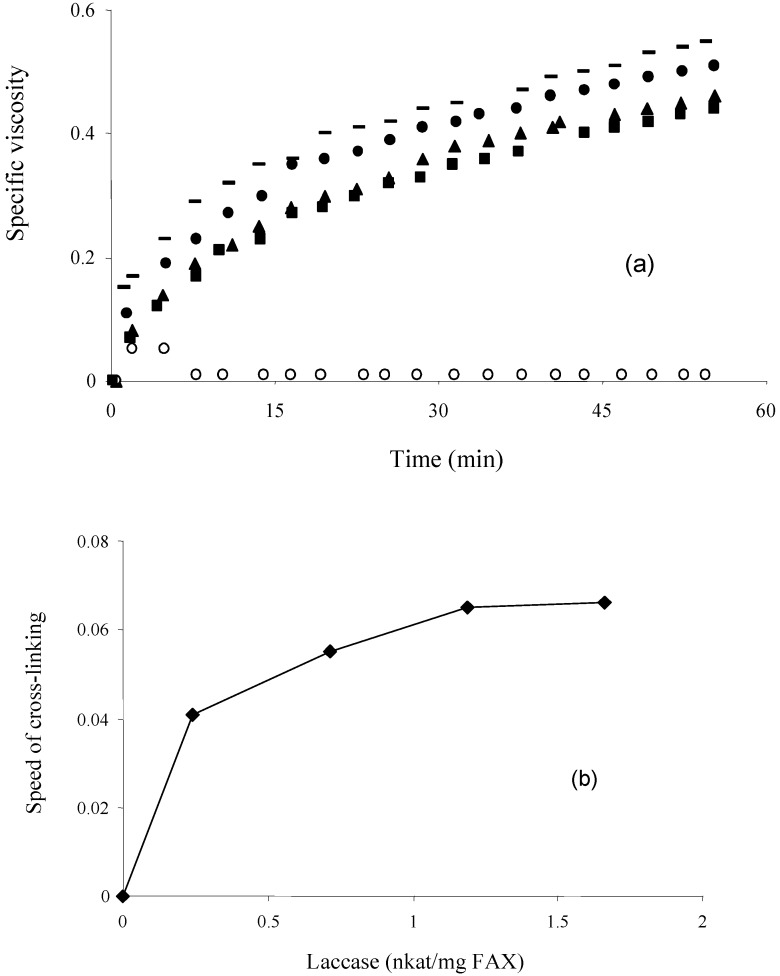
(a) Capillary viscosimetry profiles of ferulated arabinoxylans solutions at 0.5% (w/v) treated with different amounts of laccase 0 (○), 0.2 (■), 0.7 (▲), 1.2 (♦) and 1.7 (–) nkat/mg ferulated arabinoxylans. (b). Rate of cross-linking of ferulated arabinoxylans as a function of laccase content. Measurements at 25 °C in 0.05 M citrate-phosphate buffer pH 5.

**Table 1:** Phenolic acids content of ferulated arabinoxylans at 2% (w/v) before and after cross-linking by a laccase from *Trametes* sp.

Phenolic acids (µg/mg ferulated arabinoxylans)	Before gelation	After gelation
FA	0.34 ± 0.01	0.06 ± 0.001
di-FA	0.77 ± 0.01	0.020 ± 0.001
tri-FA	0.39 ± 0.001	0.01 ± 0.001

All results are obtained from triplicate runs.

## 3. Experimental

### 3.1. Materials

A wild strain of the basidiomycete *Trametes* sp. grown under drought conditions was collected from deadwood in Northern Mexico (28° 28’ N, 107° 16’ W and 2,070 m altitude). The fruiting bodies were first rinsed to remove soil residues, then treated with 10% (v/v) hypochlorite for 5 minutes, afterwards with ethanol 50% (v/v) for 5 minutes, and finally rinsed with sterilized distilled water. Then fruiting bodies were dissected in cross-sectional and longitudinal fashion to get spores from different points. These cuts were planted on potato dextrose agar (PDA) and incubated at 27 °C for a period of 7 days. Maize ferulated arabinoxylans were obtained and characterized as previously described [14]. Ferulated arabinoxylans presented a ferulic acid, di-FA and tri-FA content of 0.34, 0.77 and 0.39 μg/mg of ferulated arabinoxylans, respectively. The relative percentages of each di-FA structure were: 16, 21 and 63% for the 8-5´, 8-*O*-4´ and 5-5´ structures, respectively. All chemical products were purchased from Sigma Chemical Co. (St Louis, MO, USA).

### 3.2. Methods

#### 3.2.1. Culture conditions

Five pieces of mycelium of approximately 1 cm^2^ were used in 250 mL of medium (10 g sucrose, 5 g yeast extract, 0.2 g MnSO_4_ and 2.0 g KHPO_4_ per liter). CuSO_4_^.^5 H_2_O 0.06 Mm was used as the laccase inductor, and the inoculated medium was incubated at 160 rpm and 25 °C in darkness. 

#### 3.2.2. Laccase activity

The supernatant was assayed for laccase activity at 25 °C by mixing laccase supernatant (0.5 mL) with 0.05 m citrate-phosphate buffer pH 5 (2.2 mL) and syringaldazine in methanol (0.2 mL, 0.216 mM). The enzymatic reaction was followed during 2 minutes at 530 nm at 25 °C with an extinction coefficient of 65 mM^−1^ cm^−1^. One unit of laccase was defined as the amount of enzyme required to cause a change of 0.1 per minute at 25 °C.

#### 3.2.3. Protein content

The protein concentration in the supernatant was determined by the method of Bradford [15], using bovine serum albumin (BSA) as standard. 

#### 3.2.4. Gelation kinetics

Laccase induced ferulated arabinoxylans gelation was followed by viscosity measurements performed at 25 °C with an automatic AVS 400 capillary viscosimeter (Schott Geräte, Hofheim, Germany), equipped with an Oswald capillary tube (0.05 m citrate phosphate buffer flow time, 28.92 s). The flow times of 2 mL of 0.5% (w/v) ferulated arabinoxylan solution were measured immediately after mixing with laccase at different concentrations 0, 0.2, 0.7, 1.2 and 1.7 nkat/mg ferulated arabinoxylan).

#### 3.2.5. Cross-linking of ferulated arabinoxylans

Ferulated arabinoxylan solution (2%, w/v) was prepared in 0.05 m citrate phosphate buffer at pH 5. Laccase (1.675 nkat/mg ferulated arabinoxylans) was used as cross-linking agent. The gel state was estimated by visual inspection of the reaction mixture after 4 h at 25 °C. The system was considered as gelled when it did not deform under its own weight when the vessel containing the reaction mixture was tilted. 

#### 3.2.6. Arabinoxylan gel cross-linking content

Phenolic compound (ferulic acid, di-FA and tri-FA) contents in arabinoxylan gels were quantified by HPLC as previously reported [9,16]. A Supelcosil LC-18-DB column (250 × 4.6 mm, Supelco, Inc., Bellefont, PA, USA) was used. Detection was by UV absorbance at 320 nm. Isocratic elution was performed using methanol/water/acetic acid (40/59/01) at 0.6 mL/min at 35 °C. A Varian 9012 photodiode array detector (Varian, St. Helens, Australia) was used to record the ferulic acid spectra. A Star Chromatography workstation system control version 5.50 was used.

### 3.3. Statistical analysis

All measurements were made in triplicates and the coefficients of variation were lower than 8%. Results are expressed as mean values.

## 4. Conclusions

*Trametes* sp. grown under drought conditions was able to produce and excrete the enzyme laccase. The laccase extracted, successfully cross-linked the ferulated arabinoxylans as evidenced by an increase in specific viscosity, the formation of di-FA and tri-FA covalent unions and the formation of a firm arabinoxylan gel. These results indicate that *Trametes* sp. can be a novel source of biopolymers cross-linking agents like laccase. Further research in this regard is undergoing to fully assess the presence of other oxidative enzymes such as tyrosinases and peroxidases, which can also be of interest for proteins or polysaccharides cross-linking.
